# Context-adaptive based CU processing for 3D-HEVC

**DOI:** 10.1371/journal.pone.0171018

**Published:** 2017-02-09

**Authors:** Liquan Shen, Ping An, Zhi Liu

**Affiliations:** Key Laboratory of Advanced Display and System Application, Ministry of Education, Shanghai University, Shanghai, China; Kaohsiung Medical University, TAIWAN

## Abstract

The 3D High Efficiency Video Coding (3D-HEVC) standard aims to code 3D videos that usually contain multi-view texture videos and its corresponding depth information. It inherits the same quadtree prediction structure of HEVC to code both texture videos and depth maps. Each coding unit (CU) allows recursively splitting into four equal sub-CUs. At each CU depth level, it enables 10 types of inter modes and 35 types of intra modes in inter frames. Furthermore, the inter-view prediction tools are applied to each view in the test model of 3D-HEVC (HTM), which uses variable size disparity-compensated prediction to exploit inter-view correlation within neighbor views. It also exploits redundancies between a texture video and its associated depth using inter-component coding tools. These achieve the highest coding efficiency to code 3D videos but require a very high computational complexity. In this paper, we propose a context-adaptive based fast CU processing algorithm to jointly optimize the most complex components of HTM including CU depth level decision, mode decision, motion estimation (ME) and disparity estimation (DE) processes. It is based on the hypothesis that the optimal CU depth level, prediction mode and motion vector of a CU are correlated with those from spatiotemporal, inter-view and inter-component neighboring CUs. We analyze the video content based on coding information from neighboring CUs and early predict each CU into one of five categories i.e., DE-omitted CU, ME-DE-omitted CU, SPLIT CU, Non-SPLIT CU and normal CU, and then each type of CU adaptively adopts different processing strategies. Experimental results show that the proposed algorithm saves 70% encoder runtime on average with only a 0.1% BD-rate increase on coded views and 0.8% BD-rate increase on synthesized views. Our algorithm outperforms the state-of-the-art algorithms in terms of coding time saving or with better RD performance.

## 1. Introduction

In the recently years, 3D video has undergone a rapid development from the release of 3D film and 3D video game to the emergence of 3D services such as stereoscopic 3DTV and free viewpoint television (FTV). However, 3D video has not yet met its expected success mainly due to the burden of wearing 3D glasses and viewing discomforts such as headaches, seizures and eyestrain. Auto stereoscopic viewing is fully prospective for the entertainment industry, which requires more views to be displayed. A new format for 3D scene representation, commonly known as multi-view video plus depth (MVD) format, is introduced by Moving Picture Experts Group (MPEG), where 2D texture videos and their corresponding per-pixel depth maps are used to represent 3D scene [1]. This kind of scene representation enables a receiver to generate virtual views through the depth image based rendering (DIBR) technique. MPEG issued a call for proposals (CfP) for MVD coding technologies. Following the response of the CfP, a joint collaborative team between ISO and ITU, called JCT-3V, has been formed, which focuses on developing a 3D extension of HEVC (3D-HEVC), after a first standardization activity finalized with multi-view video coding.

**Fig 1:**
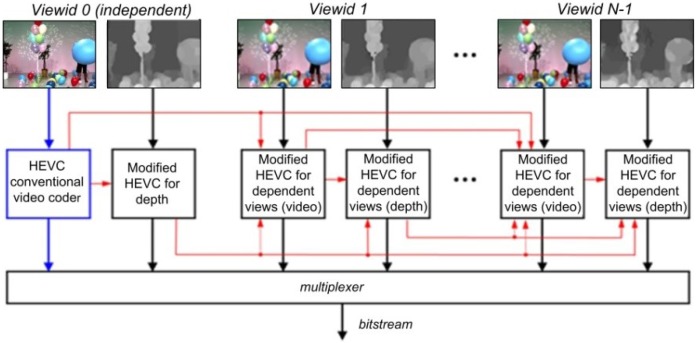
Coding structure of 3D-HEVC.

3D-HEVC aims at coding of 3D videos that usually contains multi-view texture data and its corresponding depth information. It inherits the same quadtree coding structure of HEVC for both texture videos and depth maps. The inter-view prediction is also applied to each view in 3D-HEVC, which uses the variable-sized prediction techniques of HEVC to exploit the inter-view correlation between neighboring views. Meanwhile, it also exploits redundancies between texture videos and its associated depth using inter-component coding tools. The basic structure of 3D-HEVC is shown in Fig 1, where the texture video and depth map corresponding to a particular camera position are marked by a view identifier ("Viewid"). The "Vieweid" is also used for specifying the coding order. The view with "Viewid" 0 is referred to as the independent view or the base view, which is coded independently of the other views using a conventional HEVC encoder. The other views are referred to as dependent views. In addition to the technologies defined in the HEVC standard, they can be coded with additional inter-view prediction tools to further improve the rate-distortion (RD) coding efficiency [2].

HEVC employs a quad-tree coding block partitioning structure that enables a flexible use of large and small coding, prediction, and transform blocks. In the current reference encoder of HEVC (HM), pictures are divided into a sequence of treeblocks, and each treeblock can be further split into so-called coding units (CU). The CU concept allows a treeblock recursively subdividing into four square blocks. This dividing process generates an adaptive coding tree structure allowing CUs that may be as large as a treeblock or as small as the minimum prediction block size (8×8 pixels) [3]. Four CU depth levels, namely 64×64, 32×32, 16×16 and 8×8, are usually supported in HEVC.

For a CU in one depth level, it can be split into 2 or 4 prediction units (PUs). PU sizes for intra coded CUs include 2N×2N and N ×N, whereas PU sizes for inter coded CUs are with 2N×2N, 2N×N, N×2N, N×N, 2N×nU, 2N×nD, nL×2N, and nR×2N. Thus, the possible prediction modes in inter frames include Inter 2N×2N, Inter 2N×N, Inter N×2N, Inter 2N×nU, Inter 2N×nD, Inter nL×2N, Inter nR×2N, Inter N×N (available for the smallest CU), SKIP mode, Merge mode, Intra 2N×2N, and Intra N×N(available for the smallest CU), [4]. The motion vectors of SKIP mode and Merge mode are directly derived from previously coded information, and thus they do not need to encode any additional motion data. Similar to the test model of HEVC, the mode decision process in the test mode of 3D-HEVC (HTM) is performed using all the possible prediction modes and CU depth levels to find the one with the minimum RD cost using RD optimization techniques [5]. The cost for mode decision *J*_*RDO*_ is specified by the following formula,

$$J_{RDO}\;={\text{(SSE}}_{luma}+\omega_{\text{chroma}}\times {\text{SSE}}_{\text{chroma}}\;)+\lambda \times \text{B}$$ (1)

where *B* denotes the bitrate cost, which depends on each decision case, *SSE* is the sum of squared error between the current CU and the matching block, *λ* is the Lagrange multiplier and *ω*_*chroma*_ is the weighing factor for chroma components.

Both CU depth level decision and prediction mode decision require heavy computational complexity for a CU. The complexity of the encoding processes becomes a real issue especially when a real time encoding of high definition videos (4K and 8K) is required. Furthermore, since the inter-view prediction is applied to every dependent view, the prediction part consisting of variable-sized motion estimation (ME) and disparity estimation (DE) becomes the most computationally intensive part in a 3D-HEVC system [5]. Therefore, fast CU processing algorithms are of critical importance.

This paper is organized as follows. Section 2 reviews related works on fast 2D/3D encoding methods, and Section 3 analyzes coding information (including prediction mode and CU depth level) correlation among inter-views/inter-components in the prediction structure of 3D-HEVC. A fast and efficient CU processing algorithm is proposed in Section 4. Experimental results and conclusions are given in Sections 5 and 6, respectively.

## 2. Overview of related works on fast encoding methods

Recently, several algorithms have been proposed to reduce the complexity of 3D video coding based on H.264/AVC, i.e., low-complexity multi-view video coding (MVC) and fast depth map coding algorithms. Studies on reduction of computation complexity of MVC are reported as follows. The fast MVC method proposed in [6] jointly uses an adaptive prediction structure and hierarchical mode decision, and fast inter mode decision is proposed in [7] based on textural segmentation and correlations of multi-view video coding. The scalable massively parallel fast search [8] is designed for MVC to significantly reduce the computational cost of ME/DE over the current best available full block matching and sub-optimal fast search algorithms. Another efficient ME/DE algorithm [9] reduces the computational complexity of MVC according to the characteristics of the coded block pattern and RD cost. As DE is different from ME, a fast DE algorithm [10] uses the camera geometry structure to reduce complexity of inter-view prediction. Fast DE algorithms in our previous work [11] utilize the spatial property of motion field to predict where needs DE or variable size ME. All these methods are efficient in reducing the computational complexity with small quality degradation. However, inter-component correlations are not exploited to reduce additional computations of depth map coding in MVD. To this end, fast depth coding has been proposed in [12–13] based on the coding information correlation between texture videos and depth maps. Although there are a lot of literatures to reduce complexity of MVC and depth map coding based on H.264/AVC, they in general cannot be directly applied to 3D-HEVC encoders, due to the new quad-tree coding structure of HEVC and additional inter-component coding tools adopted in 3D-HEVC.

A number of fast coding algorithms [14–24] have been proposed to reduce the complexity of state-of-the-art video coding standard, HEVC. The optimized parallel structure which is designed to speed up HEVC encoders [14] includes two levels of parallelism: frame-level and task-level. A novel coding strategy is proposed in [15] to reduce the computation complexity in HEVC encoders by efficient selection of appropriate block-partitioning modes based on human visual features (HVF), and low complexity coding tree mechanism is proposed in [16] for HEVC fast CU encoding, which makes an in-depth study of the relationship among CU distribution, quantization parameter and content change. The watermark is embedded into the Quantized Transform Coefficients in [17] during the HEVC encoding process. Later, during the decoding process, the embedded message can be detected and extracted completely. The pyramid motion divergence and markov random field are used to help selecting CU size [18], and mode filtering based on texture complexity analysis is used to reduce the number of candidates of HEVC intra prediction [19]. Non-zero transformed coefficient levels are utilized in [20] to perform early termination of the mode decision process. Early termination of CU encoding (ECU) [21] based on SKIP mode flag and encoding decision early termination (CFM) [22] based on coded block flag are proposed to speed up the mode decision procedure. Effective CU size decision and mode decision algorithms are also proposed in our previous work [23–24] to reduce the coding complexity of HEVC encoders. The aforementioned methods are well developed for HEVC encoders to achieve significant time saving. However, they are not efficient for dependent view coding in 3D-HEVC encoders since its prediction structure involves inter-view prediction and inter-component prediction, which is different from that of a conventional HEVC encoder.

We will briefly review below fast encoding methods proposed so far for 3D-HEVC. For a better representation of edges in depth maps, the new intra prediction modes, depth-modeling modes (DMMs) have been integrated into the 3D-HEVC standard [25]. Currently, some state-of-the-art work on complexity reduction focuses on reducing the complexity of depth coding in 3D-HEVC. A fast intra mode selection algorithm is proposed to speed up depth coding using most probable mode as the indicator [26]. However, coding information correlations among multi-views are not efficiently utilized. To utilize the inter-view correlation, a depth quad-tree limitation algorithm with a predictive coding of the quadtree (QTL-PC) has been proposed to reduce depth coding complexity, where a given depth CU cannot be split more than its collocated CU in the texture [27]. Furthermore, a low-complexity adaptive view synthesis optimization scheme [28] and a fast mode decision algorithm of the dependent texture videos (FMDT) [29] are proposed to accelerate mode decision. In our preliminary work, a fast mode decision algorithm (FMD) is proposed for 3D-HEVC, which incorporates two fast mode decision strategies: early SKIP mode decision and inter prediction size correlation based mode decision [30]. Fast mode decision algorithm for depth map coding utilizes the grayscale similarity and inter-view correlation in [31], where the grayscale similarity and inter-view correlation are jointly used for dependent views to achieve early decision on the best PU mode. Online-learning-based complexity reduction scheme for 3D-HEVC [32] decreases the complexity of the inter/intra mode search process of the to-be-encoded blocks in the dependent texture views, utilizing the two-mode prediction approaches, motion information of the base texture view, and the rate distortion cost of the already encoded blocks. An efficient depth modeling mode decision algorithm for 3D-HEVC depth map coding is proposed in [33], which incorporates unnecessary depth blocks skipping approach and Contour pattern early termination method. To further relieve the computation complexity of 3D-HEVC encoders, this paper proposes a context-adaptive based CU processing algorithm for 3D-HEVC encoders with CU classification to jointly optimize CU depth level decision, mode decision, ME and DE processes. The proposed approach is based on the hypothesis that the optimal CU depth level, prediction mode and motion vector in 3D-HEVC have a strong correlation with spatiotemporal, inter-view and inter-component neighboring CUs. We exploit these correlations to analyze video content and classify each CU into one of five categories: DE-omitted CU, ME-DE-omitted CU, SPLIT CU, Non-SPLIT CU and normal CU. Each type of CU adaptively adopts different processing strategies.

## 3. Observation and analysis

In HTM, a complex RD optimization (RDO) process is performed at each CU depth level to find one CU size with the minimum RD cost and determine the best CU size. Choosing smaller CU size (e.g., 8×8) usually results in a lower-energy residual after motion/disparity compensation, but consumes a larger number of bits to signal the information of coding modes and motion vectors. On the other side, choosing large CU size (e.g., 64×64, 32×32) means that a smaller number of bits are required to signal the type of coding mode and the choice of motion vectors; however, the motion/disparity compensated residual between current CU and the matching block usually contains significant amount of energy in regions containing rich texture or complex object motion. Therefore, it is sufficient to predict current CU on a coarse level for smooth image region, and coding using larger CU sizes will not result in a much larger residual. A small CU size is appropriate for regions with complex texture coming from different objects.

The CU depth level (CUDL) distribution and their correlations among multi-view videos and inter-components are first analyzed using HTM 16.0 encoders. We encode 4 sequences with different motion activities and spatial resolutions of 1024×768 ("Balloon" and "Kendo") and 1920×1088 ("PoznanStreet" and "PoznanHall2"). Among these test sequences, "Balloon" and "PoznanStreet" are with a large global/local motion, while "PoznanHall2" is with a smooth motion or a medium local motion. Test conditions are set as follows: the hierarchical B frame structure under a group of picture length equal to 8 is used; RDO enabled, and search range of ME and DE is 64; quantization parameters (QPs) are chosen with 20, 25 and 35;treeblock has a fixed size of 64×64 pixels and a maximum CU depth level of 4. The HTM16.0 original encoder without any simplifications during mode decision is used in the analysis.

Table 1 shows average CUDL distribution for texture videos and depth maps. The results show that 69%, 12%, 8% and 11% of texture treeblocks (62%, 12%, 6% and 19% of depth treeblocks) choose the CU depth level "0", "1", "2", and "3", respectively. For sequences containing a large area with complex motion such as "Ballon", the possibility of selecting the CU depth level "0" is relatively low, especially at large bitrate (QP = 20). For sequences containing a large area with homogeneous texture or smooth motion, such as "Poznanhall2", the possibility of selecting the CU depth level "3" is relatively low, with the average value of 5% at texture videos (10% at depth maps). These results show that small CU depth levels (large CU sizes) are more likely to be selected for sequences with large homogeneous area, and large CU depth levels are selected at treeblocks with high detail. The optimal CU depth level should be adaptively determined based on image content of each treeblock. We can see that 40%-75% treeblocks choose the last three CU depth levels at high bitrate (QP = 20), and thus inter/intra prediction on CU size of 64×64 can be adaptively bypassed. Meanwhile, it can be seen that most of treeblocks choose the first two CU depth levels especially for low activity sequences. 66% and 12% treeblocks choose the optimal CU depth levels with level "0" and level "1", respectively, and only about 15% treeblocks choose the last depth level (level "3") on average. Thus, inter/intra prediction on small CU sizes could be skipped in most cases without loss of coding performance.

**Table 1:** CU depth level (CUDL) distribution for texture videos and depth maps (%).

		Texture video				Depth maps			
Sequences	QP	CUDL = 0	CUDL = 1	CUDL = 2	CUDL = 3	CUDL = 0	CUDL = 1	CUDL = 2	CUDL = 3
Ballon	20	48.9	19.0	14.4	17.6	35.7	7.4	7.8	49.2
	25	62.0	19.2	9.9	9.0	58.0	20.1	11.8	10.1
	35	84.7	9.5	3.5	2.2	68.7	19.8	8.6	2.8
Kendo	20	53.3	14.8	13.1	18.7	39.7	7.2	7.2	45.9
	25	67.4	13.4	7.1	12.1	73.0	11.8	6.1	9.1
	35	79.4	11.1	4.0	5.5	76.3	15.9	4.3	3.6
Poznanhall2	20	60.1	15.1	13.2	11.6	59.9	9.6	7.4	23.1
	25	81.7	11.4	4.0	2.9	77.6	12.2	3.7	6.5
	35	91.4	6.7	1.3	0.6	78.3	17.9	3.6	0.3
Poznanstreet	20	42.0	14.7	15.9	27.3	25.4	6.7	7.8	60.1
	25	67.7	3.6	3.5	15.8	72.7	9.5	5.0	12.9
	35	86.5	1.2	2.2	3.8	81.7	10.2	4.3	3.8
Average		68.8	11.6	7.7	10.6	62.3	12.4	6.4	18.9

Table 2 shows average prediction mode distribution for texture videos and depth maps under different QPs (20, 25 and 35). It can be observed that most of CUs in inter frames choose SKIP mode as the optimal prediction mode, and Merge mode achieves second high percentage to be chosen as the best inter mode. The total percentage of SKIP mode and Merge mode reaches 87% for texture videos and 90% for depth maps, while the total percentage of other modes is about 13% for texture videos and 10% for depth maps. Especially, for low motion sequences such as "Poznanhall2", more than 94% of CUs in depth maps are coded with SKIP mode. The reason is that stationary regions and smooth motion regions often exist in natural video sequences and depth maps, and these two classes of regions are more likely to select SKIP mode and Merge mode. If we can pre-determine for a CU whether the optimal prediction mode is SKIP mode or Merge mode, the process of variable-sized ME and DE can be bypassed, and thus a huge amount of computation complexity can be reduced. The total probability for a CU to be coded with small and medium-sized inter modes (including inter 2N×N, inter N×2N, inter N×N, inter 2N×nU, inter 2N×nD, inter nL×2N, inter nR×2N) and intra modes is very low, which is less than 7% for depth maps and 11% for texture videos. Table 2 also shows the prediction mode distribution at each CU depth level. It can be seen that CUs at higher depth levels are more likely to choose SKIP mode and Merge mode. At CU depth level "3", the percentage of SKIP mode reaches 94% and 97% for texture videos and depth maps, respectively.

**Table 2:** Prediction mode distribution for depth maps and texture videos (%).

		Texture video							Depth map						
	CUDL	SKIP	Merge	Inter 2Nx2N	Inter 2NxN	Inter Nx2N	Other inter	Intra modes	SKIP	Merge	Inter 2Nx2N	Inter 2NxN	Inter Nx2N	Other inter	Intra modes
Ballon	0	72.2	3.5	2.8	7.5	7.9	5.7	0.4	77.8	6.4	3.8	2.6	2.7	1.5	5.3
	1	84.3	4.1	1.8	2.4	2.8	4.0	0.6	86.0	2.2	2.9	1.4	1.4	2.0	4.2
	2	91.4	3.5	1.3	0.8	1.1	1.1	0.8	92.6	0.7	2.1	0.9	0.6	0.8	2.4
	3	95.7	2.3	0.6	0.3	0.5	0.0	0.6	96.7	0.1	1.4	0.2	0.3	0.0	1.3
Kendo	0	68.2	4.3	4.5	6.9	8.7	5.6	1.8	67.4	5.1	4.7	2.6	2.4	1.7	16.1
	1	80.7	5.7	2.1	2.4	2.8	4.2	2.1	79.3	1.9	3.7	1.4	1.4	2.3	10.1
	2	88.9	4.3	1.3	0.8	1.1	1.3	2.3	89.1	0.7	2.7	0.9	0.6	0.9	5.1
	3	93.8	2.7	0.5	0.9	1.3	0.0	0.8	95.0	0.2	1.7	0.3	0.3	0.0	2.6
Poznanhall2	0	73.2	1.5	6.3	4.1	8.8	3.3	2.8	82.0	1.5	4.6	1.9	2.7	0.9	6.4
	1	84.1	2.3	2.8	1.5	2.9	3.5	2.9	91.1	0.7	2.5	0.6	0.7	0.9	3.5
	2	91.0	2.1	1.5	0.5	1.1	1.2	2.6	96.4	0.3	1.0	0.2	0.2	0.3	1.7
	3	95.3	1.4	0.8	0.2	0.5	0.0	1.8	98.6	0.1	0.4	0.0	0.1	0.0	0.8
Poznanstreet	0	65.3	4.0	3.5	10.4	8.8	6.9	1.1	85.2	4.9	2.4	1.6	1.6	1.4	2.9
	1	75.2	4.7	2.6	4.6	4.3	7.3	1.3	89.8	2.2	2.1	0.8	0.8	1.4	2.9
	2	83.4	5.3	2.0	2.2	2.2	3.3	1.6	94.1	1.0	1.6	0.5	0.4	0.6	1.8
	3	89.4	4.8	1.2	1.2	1.5	1.9	1.9	97.1	0.4	1.0	0.2	0.2	0.0	1.1
Average		83.3	3.5	2.2	2.9	3.5	3.1	1.6	88.6	1.8	2.4	1.0	1.0	0.9	4.3

**Fig 2:**
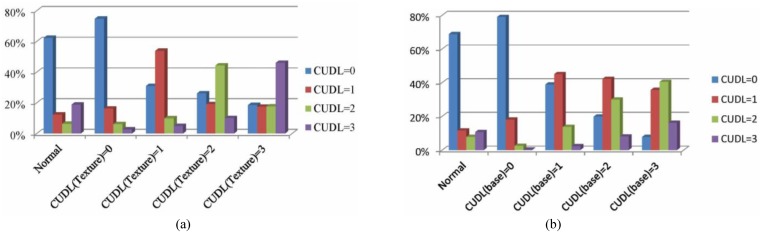
CU depth level correlation between inter-components/ inter-views. (a) Conditional probability of the optimal CUDL for current CU (in depth maps) based on the CUDL of CU_texture_; (b) Conditional probability of the optimal CUDL for current CU (in texture videos) based on the CUDL of CU_base_; "Normal" denotes the average distribution of the whole videos.

The depth map is used to represent, with other magnitude, the same scene captured by the corresponding texture cameras; therefore, some similarities in terms of CU depth level can be found in texture videos and depth maps. Fig 2(a) illustrates the percentage of the CU depth level of the co-located CU at texture videos (CU_texture_) to be the optimal CU depth level for current CU at depth maps. We can find from statistic results in Fig 2(a) that the CU depth level of CU_texture_ possesses a large ratio to be the best depth level of a CU in depth maps. For example, when CU_texture_ is coded with the CU depth level "0", the current CU is unlikely to choose other CU depth levels (the total percentage of other CU depth levels is less than 25%). This tendency indicates that the coding information of texture videos and its depth maps has similarities that can be exploited.

Content similarities can be found in multi-view videos, which lead to a strong correlation of coding information between different views. Fig 2(b) shows the optimal CU depth level correlation between current CU and its corresponding CU in the neighboring view. The corresponding CU is located by means of the neighbor disparity vector (NBDV) adopted in HTM, which is derived from several spatial and temporal neighboring CUs [34]. When the corresponding CU in neighbor views (CU_base_) chooses the optimal CU depth level "0", the average probability of current CU choosing the CU depth level "0" reaches 79% or more, much larger than the average distribution of the whole videos (the normal one in Fig 2(b)). When CU_base_ chooses level "1", "2" or "3", the current CU tends to significantly increase the percentage of level "1", "2" or "3" compared to the normal case. Thus, CU depth level or prediction mode in one view could be decided according to the corresponding one in the neighboring views.

Meanwhile, strong spatial correlation can be found in natural texture videos and depth maps, especially in the smooth texture regions. The neighboring CUs experience nearly the same motion, which are with similar motion vectors, the optimal CU depth level and coding mode. Most CUs do not exhibit a wide difference from the co-located CU in the previous frame due to high correlations among successive frames, and coding information of the co-located CU from previous frames affects the CU depth level/mode determination process of current CU. Thus we can exploit coding information from spatially/temporally adjacent CU to describe motion/texture properties of current CU and discard intra/inter prediction on unnecessary CU depth levels.

**Fig 3:**
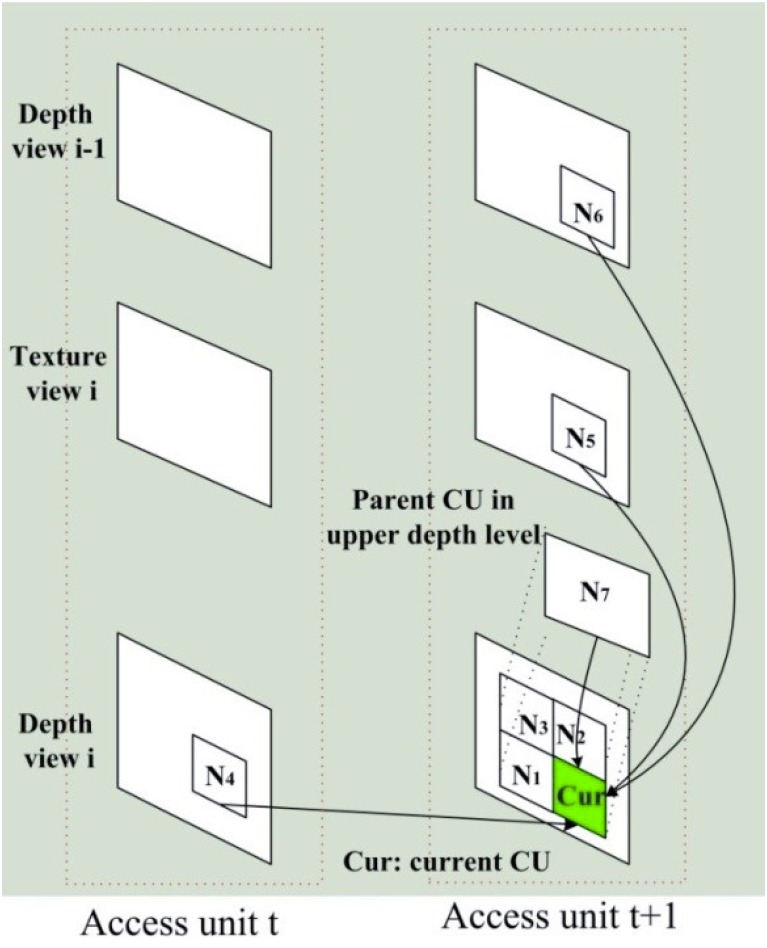
Predictors and current CU.

To code the current CU, the already coded data from neighboring CUs are used to remove unnecessary computation. A set of predictors (∅) is defined in Eq 2,

$$\emptyset =\left\{P_{{}_{1}},P_{{}_{2}},P_{{}_{3}},P_{{}_{4}},P_{{}_{5}}\right\}$$ (2)

where *P*_1_ is spatial predictors (including *N*_1_, *N*_2_ and *N*_3_ in Fig 3), *P*_2_ is the temporal predictor (*N*_4_ in the temporal reference frames) located at the same position as the current CU (*C*_*cur*_), *P*_3_ and *P*_4_ respectively denote the inter component predictor (*N*_5_) and the corresponding CU from neighbor coded views(*N*_6_), and *P*_5_ is the parent CU (*N*_7_) at the upper depth levels.

In our algorithm, CUs are classified into five classes including DE-omitted CU, ME-DE-omitted CU, SPLIT CU, Non-SPLIT CU and normal CU. Specifically, 1) a DE-omitted CU is coded based on ME, and does not need to require DE; 2) a ME-DE-omitted CU is coded with either SKIP or Merge mode, and does not require ME and DE; 3) a SPLIT CU prefers to be directly divided into sub-CUs, and does not need intra and inter prediction on the current CU size; 4) a Non-SPLIT CU is predicted well with the current CU size, and does not need to be divided into sub-CUs; 5) if a CU does not belong to the above four classes, it is defined as the normal CU. According to coding information from the predictor set, ∅, each CU is pre-determined as one of the above five classes and different processing strategies are adopted based on the pre-determined class of CU.

## 4. Classification based fast CU processing for 3D-HEVC

### 4.1 Early DE-omitted CU determination

As mentioned in the Section 1, DE is used to exploit inter-view dependence, and the process of disparity search finds the best prediction block from neighboring coded views. Although motion-compensated prediction is generally the most efficient prediction mode in multi-view video coding, the analysis of motion-compensated prediction and disparity-compensated prediction in [35] indicates that using both motion-compensated prediction and disparity-compensated prediction can improve coding efficiency. The reason is that temporal motion cannot be characterized adequately for non-rigid motion object or motion boundaries. Almost all video coding standards use block-based motion estimation and compensation, which is based on simple translation movement. It fails for non-rigid motion, produces large magnitude of motion vectors and higher residual coefficients in motion boundary region and thus leads to a poor prediction. Therefore, the regions with smooth motion are more likely to choose motion-compensated prediction, and regions with complex motion are more likely to choose disparity-compensated prediction.

The motion vectors of neighboring coded view and those of the current views are correlated. In our algorithm, we utilize this correlation to adaptively enable DE for the dependent view based on analyzing motion data of the corresponding coded treeblock in base view located by the "DoNBDV" tool. Assume that the corresponding treeblock in the neighboring coded view located at the *m*th row and *n*th column is denoted as *TB*_*m*,*n*_, and motion vectors (MVs) of its covered N×N blocks (Here, *N* = 16) are thus denoted as *mv*_*i*,*j*_ = {*mvx*_*i*,*j*_, *mvy*_*i*,*j*_}, *i* ∈ [4*m*, 4*m* + 3], *j* ∈ [4*n*, 4*n* + 3]. The mean deviation of MVs is calculated based on Eq 3 by exploring 16 motion vectors covered by *TBm*,*n*. The mean deviation (MD) of MVs in Ω, is defined as

$$MD(\Omega )=\frac{1}{k}\cdot \sum_{\Omega }\left\{\left|mvx_{i,j}-\frac{1}{k}\cdot \sum_{\Omega }mvx_{i,j}\right|+\left|mvy_{i,j}-\frac{1}{k}\cdot \sum_{\Omega }mvy_{i,j}\right|\right\}$$ (3)

Ω represents the region covered by the corresponding coded treeblock in base view, and *k* is the total number of blocks in Ω. When the mean deviation is smaller than a threshold *Ts*, current CU is considered with homogeneous motion and early determined as the DE-omitted CU. The threshold *Ts* is set to 0.1 in order to tolerate one noisy MV in the motion homogenous region. The noisy MV is different from the other *k* − 1 MVs by only one 1/4-pel in either horizontal or vertical direction, and *Ts* is selected to strictly satisfy the above limitation.

**Fig 4:**
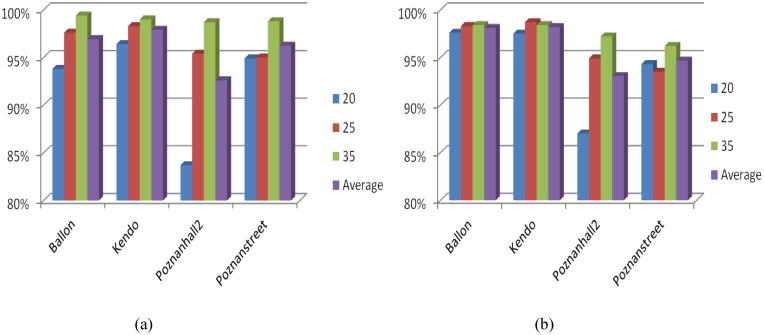
Accuracy of the proposed algorithm to early determine the DE-omitted CUs. (a) texture video; (b) depth maps.

To analyze the efficiency of the proposed method, we calculate the accuracy of the proposed algorithm to early determine the DE-omitted CU in Fig 4 utilizing the exhaustive mode decision in HTM under the aforementioned test conditions in Section 3. It can be seen that the average accuracy of the proposed algorithm reaches 96% on both texture videos and depth maps with the maximum of 99%. The results shown in Fig 4 indicate that the proposed method can accurately reduce unnecessary DE on CUs with homogenous motion.

### 4.2 Early ME-DE-omitted CU determination

An inter-frame CU in 3D-HEVC can be coded with one of the following coding modes: SKIP mode, Merge mode, normal inter prediction modes and intra modes. Normal inter modes include inter 2N×2N, inter 2N×N, inter N×2N, inter N×N, inter 2N×nU, inter 2N×nD, inter nL×2N and inter nR×2N. When a CU is coded with SKIP mode, the CU is associated with one PU and has no significant residual coefficients, no coded motion vector delta or reference picture index. A Merge mode is specified whereby the motion parameters for the current PU are obtained from neighboring PUs, including spatial and temporal candidates. Merge mode and SKIP mode cost little computational complexity since they do not need ME and DE. Meanwhile, they achieve good coding efficiency. There are substantial amounts of background and static regions in normal video sequences, which are quite suitable for coding in SKIP mode and Merge mode. Therefore, if Merge mode and SKIP mode can be early determined, ME and DE on all PUs can be omitted. Thus, significant coding time could be reduced.

Assume the adjacent predictors of current CUs (*Cur*) to be P_i_, i = 1, 2, 3, 4, 5 in Eq 2, we can predict the mode of the current CU (mode(Cur)) based on the relation between the adjacent CUs and *Cur* as follows,

$$\text{mode}(\text{Cur})\in \{\begin{array}{c} \text{SKIP/Merge, }\sum_{\text{i}=1}^{5}{\gamma (\text{mode}(\text{P}}_{\text{i}}))>1\;\\ \text{non(SKIP/Merge), others } \end{array}$$ (4)

where γ(mode(P_i_)) is the characteristic function denoted by

$${\gamma (\text{mode}(\text{P}}_{\text{i}}\text{)})\;=\;\{\begin{array}{c} {1\text{, mode}(\text{P}}_{\text{i}})\in \text{SKIP/Merge}\\ \text{0, others } \end{array}$$ (5)

**Fig 5:**
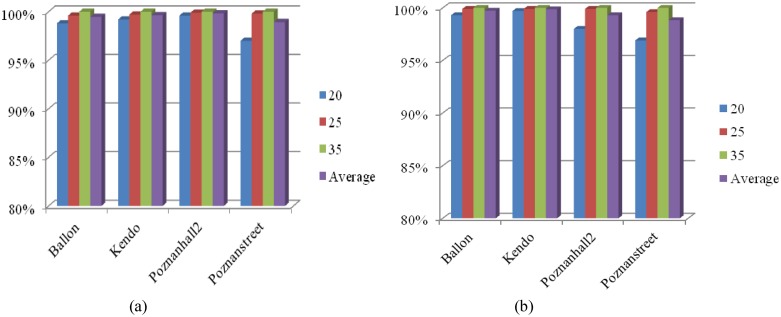
Accuracy of the proposed algorithm to early determine ME-DE-omitted CUs. (a) Texture videos; (b) Depth maps.

When the prediction mode of current CU is determined as the "SKIP/Merge" mode, we early determine it as a ME-DE-omitted CU. By exploiting the exhaustive mode decision in HTM under the aforementioned test conditions in Section 3, we calculate the accuracy of the proposed algorithm to pre-determine the ME-DE-omitted CU in Fig 5. As shown in Fig 5, the average accuracy of our algorithm to detect ME-DE-omitted CUs achieves more than 99.5% on both texture videos and depth maps with a minimum of 97%. Furthermore, it is consistently high for all test sequences with different activities and different configurations (QPs). If the encoder can decide the ME-DE-omitted CU at the early stage, the procedures of ME/DE and mode decision can be early terminated, and coding time can be reduced dramatically.

### 4.3 Early SPLIT CU determination

In order to reduce the computational complexity, the proposed method allows skipping the full RD cost computation for a CU, which directly divides into sub-CUs. If it is determined in advance that a CU is a SPLIT CU, intra prediction and inter prediction on current CU size are not necessary. It can be seen from analysis in Section 3 that the quad-tree of a treeblock is linked to those from spatial, temporal, inter-view and inter-component neighboring treeblocks. We can make use of the optimal CU depth level from neighboring CUs to predict the optimal CU depth level of current CU and perform early determination of the SPLIT CU. The optimal CU depth level of current CU (CUDL) is predicted as follows,

$$CUDL_{pred}=\frac{\sum_{i=1}^{M}k_{i}\cdot w_{i}\cdot CUDL_{i}}{\sum_{i=1}^{M}k_{i}\cdot w_{i}}$$ (6)

where *M* is the number of neighboring CUs in *P*_1_, *P*_2_, *P*_3_ and *P*_4_ (*N*_*i*_, i = 1, 2, 3…6 in Fig 3) equal to 6, *CUDL*_*i*_ is the actual CU depth level selected for coding of the neighboring CU, *N*_*i*_. Only available adjacent CUs in ∅ will be used. Hence, *k*_*i*_ is set to 1, when *N*_*i*_ is available.

*w*_*i*_ is the CU-weight factor, which is assigned to the adjacent CUs according to their correlation from the current CU. The stronger correlation between the neighboring CU in ∅ and current CU, the larger the weight should be assigned. Meanwhile, CU-weight factors also satisfy the sum one property, $\sum_{i=1}^{M}w_{i}=1$. Therefore, *w*_*i*_ is calculated based on the Pearson's correlation coefficient (*ρ*) of the optimal CU depth level correlation between current block and each neighboring block as follows,

$$w_{i}=\frac{\rho_{i}}{\sum_{i=1}^{M}\rho_{i}}.$$

Correlation coefficient represents the degree of correlation of the neighboring blocks. If it has a large value, it means that two blocks have a consistent optimal CU depth level and there is a high possibility that two blocks have the same CU depth level. The formula for (*ρ*) is,

$$\rho_{\text{X,Y}}\;=\;\frac{\text{cov }(\text{X, Y})}{\sigma_{\text{X}}\cdot \sigma_{\text{Y}}}$$ (7)

where *X* and *Y* represent the optimal CU depth level distributions of the current CU and its neighboring CU, respectively. cov is the covariance, and *σ* is the standard deviation. In order to cope with different characteristics of video sequences, the correlation coefficients are calculated based on results of the original 3D-HEVC coding process where the proposed fast algorithm is not applied. Actually, the first inter frame in a group of coding sequence is used a training frame for the online update and encoded using the original HTM encoders, while the successive frames in the group are coded by the proposed algorithm. Meanwhile, the group length should not be too short, since the training frame will employ full prediction mode and depth level searching which will lead to complexity increase. In practice, the group length equal to 32 is used to balance the complexity of the training process and the coding performance.

**Fig 6:**
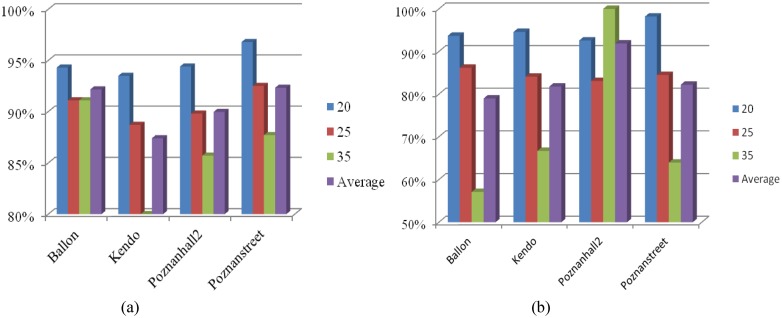
Accuracy of the proposed algorithm to pre-determine the SPLIT CU. (a) texture video; (b) depth map.

When current depth level is smaller than the predicted optimal depth level (based on Eq 6) minus 1, current CU may be composed by complex contents from different objects. It has a high probability to be coded with smaller CU size, which is considered as a SPLIT CU. RD cost computation of each prediction mode can be skipped on current CU size. Extensive simulations have been conducted on four video sequences to show the accuracy of the proposed algorithm to early determine the SPLIT CU. By exploiting the exhaustive CU depth level decision in HTM using test sequences and test condition in Section 3, Fig 6 shows that the average accuracy to early determine the SPLIT CU is about 91% on texture videos and 84% on depth maps, which is consistently high for all QPs and test sequences with different properties. Fig 6 verifies the rationality of the proposed method to early detect SPLIT CU, and inter prediction and intra prediction can be accurately bypassed for SPLIT CUs.

### 4.4 Early Non-SPLIT CU determination

It can be seen from Section 3 that most of optimal CU depth levels after the mode decision procedure are selected as either "0" or "1", especially in large QPs. CU splitting and inter/intra prediction on small CU size would be unnecessary in some cases. Therefore, it is better to introduce a Non-SPLIT CU early determination method into the CU size decision procedure. The main idea of the proposed method is to use coding information from neighboring blocks to check whether the prediction mode and motion vectors (MVs) on current CU size can represent motion efficiently or not and whether it is necessary to perform prediction on the next CU depth level. In the following, we will detail three criterions to early determine the Non-SPLIT CU. For a CU whose available adjacent CUs are all coded with CU size larger than or equal to current CU size, we consider it as a potential Non-SPLIT CU. When it simultaneously satisfies one of three following criterions, we early determine it as a Non-SPLIT CU.

#### Criterion1: MVs deviation checking based criterion

A lot of local smooth regions belong to the same video objects or background, which have close motions. Motion on a coarse level is sufficient to describe for those homogeneous motion regions, which are coded using larger CU sizes without larger residual coefficients. On the other side, it is necessary to represent motion in a finer level for discontinuous motion boundary. Therefore, motion homogeneity is a good indication in choosing the optimal CU size, which can be used to skip inter/intra prediction on unnecessary CU size so as to optimize the coding procedure. The deviation of MVs from the current CU and those from N×N blocks covered by spatially predictors (*N*_1_, *N*_2_ and *N*_3_) is used to evaluate the motion homogeneity. MV information of the current CU is achieved from inter prediction in the current CU depth level. Assume that MVs of the current CU and the covered N×N blocks (Here, *N* = 16 or *N* = 8) by neighboring CUs are denoted as *mv*_*i*,*j*_ = {*mvx*_*i*,*j*_, *mvy*_*i*,*j*_}, Ω represents regions covered by current CU and its neighboring CUs. The mean deviation of MVs is computed based on Eq 3. When the mean deviation of MVs is smaller than a threshold (*Ts*) in Section 4.1, the current CU is considered with homogeneous motion, and inter/intra prediction on sub-CU is suitable to be skipped; otherwise the CU is considered with complex motion.

#### Criterion 2: RD cost checking based criterion

3D-HEVC encoder calculates RD costs for all inter/intra modes at each CU depth level, and the one with minimum RD cost is selected. If the RD cost computed at the current CU depth level is small enough (that is, below a pre-set threshold), the current CU is probably motionless or in local homogeneous region; thus, current CU size is sufficient for accurate prediction, and the CU depth level decision process proceeding to the small CU size can be terminated. RD costs of spatially neighboring CUs (left CU, upper CU, right-upper CU, and left-upper CU) are extracted to determine the threshold for RD cost based early termination, which can adaptively change with video content. The threshold, *J*_*RDO*_(*thr*), is calculated as follows,

$$J_{RDO}(thr)=ƛ\cdot \frac{\sum_{i=1}^{N}k_{i}\cdot w_{i}\cdot J_{RDO}(i)}{\sum_{i=1}^{N}k_{i}\cdot w_{i}}$$ (8)

where *N* is the number of spatial neighboring CUs equal to 4, ƛ is an adjust parameter, *J*_*RDO*_(*i*) is the RD cost value of the neighboring CU_i_, and *w*_*i*_ is the weights determined based on the RD cost correlation between current CU and its neighboring CU_i_ as Eq 7. The selection of the parameter ƛ should keep a high accuracy while greatly reducing the complexity. Based on extensive experiments, the parameter ƛ is set to 0.5, which achieves a good and consistent performance on a variety of 3D video sequences with different activities.

#### Criterion 3: all-zero block checking based criterion

A CU whether is preferred to be coded with current CU size or be divided into sub-CUs also can be checked based on the detection of all-zero block (AZB), which represents the block with all zero coefficients after quantization. In addition, there are a substantial number of homogeneous regions with the same video content as the background. After prediction, the residuals of these homogenous regions have high probabilities to be transformed and quantized to zeros. It indicates that the motion can be efficiently represented in current CU size. Further processing of sub-CUs should be unnecessary if the resulted block-matching residuals are already quite small with all-zero after quantization.

**Fig 7:**
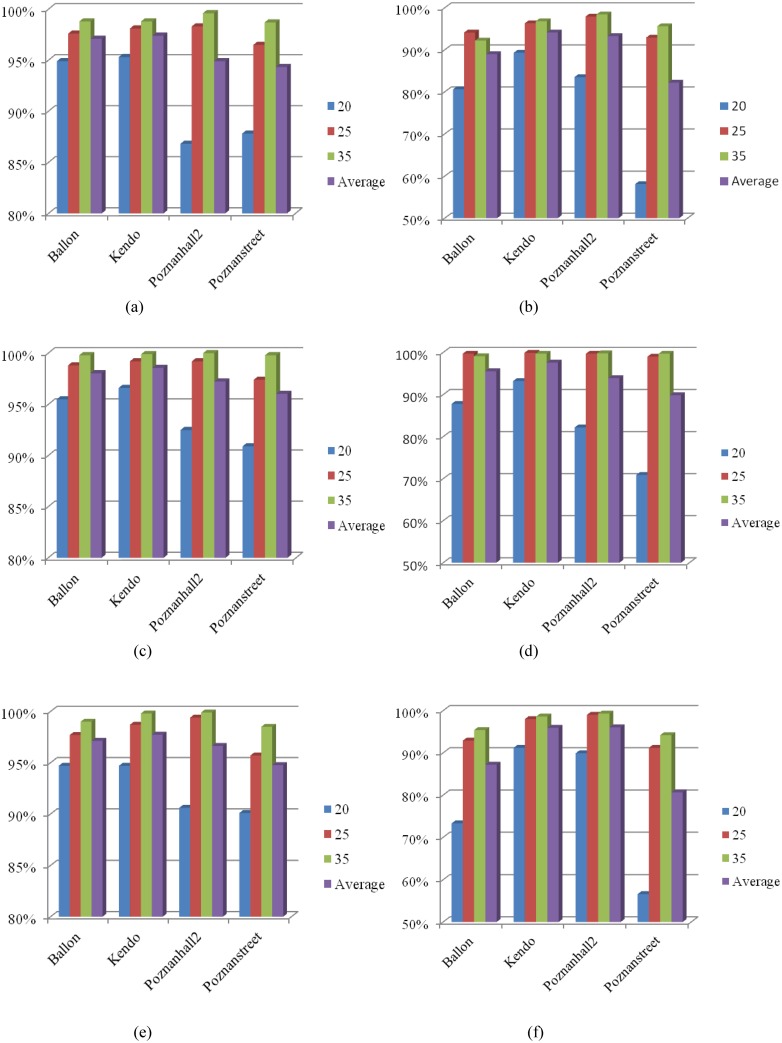
Accuracy of three proposed criterions to pre-determine the Non-SPLIT CU. (a) Criterion 1 on texture video; (b) Criterion 1 on depth map; (c) Criterion 2 on texture video; (d) Criterion 2 on depth map; (e) Criterion 3 on texture video; (f) Criterion 3 on depth map.

To verify legitimacy of three proposed criterions to early determine the Non-SPLIT CU, extensive simulations are conducted on 4 video sequences as listed in Table 1. We investigate the effectiveness of three proposed criterions by exploiting the exhaustive CU depth level decision and mode decision in HTM under the aforementioned test conditions in Section 3. Fig 7 shows the accuracy of each criterion. The average accuracy of the MVs deviation checking based criterion achieves 96% on texture videos and 90% on depth maps with a maximum of 99%. As far as the proposed RD cost checking based criterion is concerned, the accuracy is the highest with 98% on texture videos and 94% on depth map. Similar to two previous criterions, the all-zero block checking based criterion also achieves a high accuracy with 97% on texture video and 90% on depth map. The results shown in Figs 7(a)~6(f) indicate that the proposed three criterions can accurately determine the Non-SPLIT CU and reduce unnecessary intra/inter prediction on small CU sizes.

### 4.5 Flowchart of the proposed algorithm

**Fig 8:**
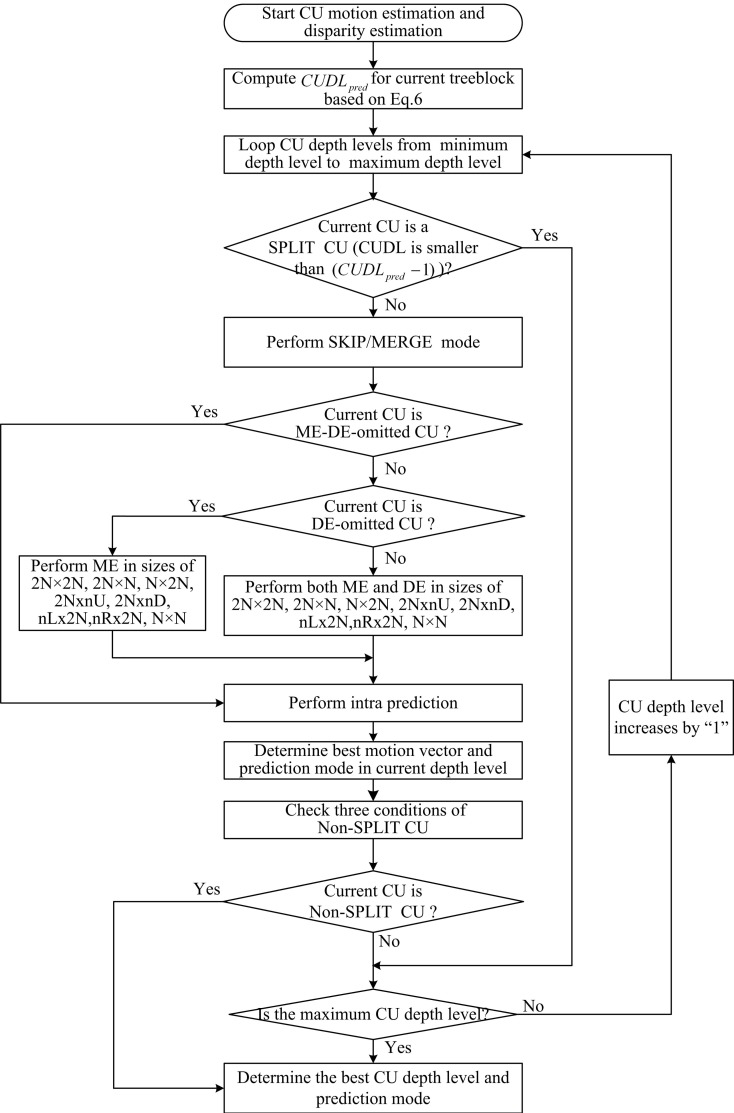
Flowchart of the proposed algorithm.

According to the above analysis, the basic idea of the proposed classification based fast CU encoding for 3D-HEVC is to adjust CU depth level decision and ME/DE processes utilizing spatiotemporal, inter-view and inter-component correlations. The coding information including optimal CU depth level, prediction mode, RD cost, and motion vectors from the predictors in ∅ are used to analyze CU property and adaptively optimize each steps of CU processing. The flowchart of the proposed fast CU processing algorithm is processed as Fig 8.

## 5. Experimental results

### 5.1 Test conditions

In order to test the performance of the proposed context-adaptive based CU processing algorithm (CACUP) for 3D-HEVC including four components, early Non-SPLIT CU determination, early SPLIT CU determination, early DE-omitted CU determination and early ME-DE-omitted CU determination, it is implemented on the 3D-HEVC reference software, HTM 16.0. The common test sequences in common test conditions (CTC) [36] for 3D-HEVC proposals evaluation are used in our experiments. The test set consists of 7 MVD sequences as tabulated in the second column of Table 3. The first three test sequences are at a resolution of 1024×768 luma/depth samples at 30fps and the last four test sequences are at a resolution of 1920×1088 luma/depth samples at 25 fps. Among these test sequences, "Balloons", "Kendo" and "PoznanStreet" are with a large global/local motion, while "Newspaper" and "PoznanHall2" are with a smooth motion. Each test sequence contains 3 texture and depth views, respectively. In CTC, the central view is usually considered as a base view and it is coded independently. Each view is composed of both texture video and depth map. The coding order is listed as follows: T0 D0, T1 D1, T2 D2 (where Ti and Di represent texture video and depth map frames in the i^th^ view). Treeblock has a fixed size of 64×64 pixels and a maximum depth level of 4, resulting in a minimum CU size of 8×8 pixels. The following QP combinations for texture video and depth map are considered in CTC: (25; 34), (30; 39), (35; 42) and (40; 45). Coding efficiency is measured with PSNR and bitrate, and computational complexity is measured with the consumed encoding time. Coding gains correspond to bitrate reductions, which are evaluated by the Bjontegaard metric (BD-rate)[37]. Positive and negative values represent increments and decrements, respectively. In Tables 4–7, the "Video PSNR/Video bitrate" (V/V) column shows the BD-rate coding results for the coded texture views. Only the texture PSNR and the texture bitrate are considered in the computation. The "Video PSNR/Total bitrate" (V/T) column shows the BD-rate coding results for coded views considered the sum of the bitrates of the three coded texture and depth views. Since the efficiency of depth map and texture video coding is improved by considering the quality of synthesizing virtual views, three synthesized views are rendered between each view after encoding. PSNRs on synthesized views are measured with respect to views synthesized using uncompressed original views. The "Synth PSNR/Total bitrate" (S/T) column shows the BD-rate results for synthesized views. The bitrate considered is the sum of the bitrates of the three coded texture and depth views. The PSNR is the average PSNR for 6 synthesized views. "ETS" column represents the percentage of runtime saving to code both texture videos and depth maps compared to the original HTM 16.0 encoder, which is computed as follow,

$$ETS=\frac{Time_{HTM}\;-\;Time_{method}}{Time_{HTM}}\times 100\%$$ (9)

where Time_method_ and Time_HTM_ denote coding time of each proposed method and the original HTM encoder, respectively. In Tables 4–7, "ETS_overall_", "ETS_texture_" and "ETS_depth_" denote time savings of the overall coding, texture coding and depth coding, respectively.

**Table 3:** Test sequences and configuration parameters.

Resolution	Test sequences	Total frames
1024×768	Balloons	300
1024×768	Kendo	300
1024×768	Newspaper	300
1920×1088	GhostTownFly	250
1920×1088	PoznanHall2	250
1920×1088	PoznanStreet	250
1920×1088	UndoDancer	250

**Table 4:** Results of the proposed algorithm compared to HTM encoders without any simplifications during mode decision.

	BD-rate result (%)						Coding time result (%)		
Test sequences	Video 0	Video 1	Video 2	V/V	V/T	S/T	ETS_overall_	ETS_texture_	ETS_depth_
Balloons	0.2	0.3	-0.1	0.2	-0.4	1.0	70	66	74
Kendo	0.5	0.2	2.1	0.9	0.7	1.0	64	62	66
Newspaper	0.2	0.1	0.1	0.2	-0.4	1.1	69	65	73
GhostTownFly	0.4	-0.4	-3.5	0.4	0.2	0.4	73	70	75
PoznanHall2	-0.1	0.7	0.5	0.5	0.4	0.7	73	70	76
PoznanStreet	0.5	-0.2	1.6	0.9	0.6	1.2	71	64	77
UndoDancer	0.2	-0.6	-1.4	0.2	-0.1	0.1	71	65	77
1024×768	0.3	0.2	0.7	0.4	0.0	1.0	68	64	71
1920×1088	0.3	-0.1	-0.7	0.5	0.3	0.6	72	67	76
Average	0.3	0.0	-0.1	0.5	0.1	0.8	70	66	74

**Table 5:** Results of the proposed algorithm compared to HTM encoder with "QTL-PC" enabled.

	BD-rate result (%)						Coding time result (%)		
	Video 0	Video 1	Video 2	V/V	V/T	S/T	ETS_overall_	ETS_texture_	ETS_depth_
Balloons	0.2	0.2	-0.1	0.1	0.0	0.2	52	60	20
Kendo	0.2	0.7	0.7	0.4	0.4	0.2	46	55	15
Newspaper	0.1	0.0	0.2	0.1	0.1	0.1	51	58	19
GhostTownFly	0.3	-0.6	-1.0	0.2	0.2	0.3	52	63	18
PoznanHall2	-0.2	0.6	0.4	0.3	0.3	-0.2	57	63	22
PoznanStreet	0.0	0.0	1.0	0.2	0.2	0.0	50	57	25
UndoDancer	0.1	-0.7	-0.9	-0.1	-0.1	0.1	51	58	25
1024×768	0.2	0.3	0.3	0.2	0.2	0.2	50	58	18
1920×1088	0.1	-0.2	-0.1	0.2	0.1	0.1	53	60	22
Average	0.1	0.0	0.0	0.2	0.2	0.1	51	59	20

**Table 6:** BD-rate performance compared with the state-of-the-art fast 3D-HEVC algorithms (%).

	CACUP		FMDT[29]		FMD[30]		QTL[27]		ECU[20]		CFM[21]		Fast HTM	
	V/V	S/T	V/V	S/T	V/V	S/T	V/V	S/T	V/V	S/T	V/V	S/T	V/V	S/T
Balloons	0.2	1.0	0.0	0.0	0.1	1.5	0.1	6.0	0.4	2.7	0.2	0.8	-0.1	6.2
Kendo	0.9	1.0	0.0	0.0	0.5	0.7	-0.1	0.7	1.7	1.8	0.5	0.5	0.1	1.0
Newspaper	0.2	1.1	0.1	-0.1	0.5	2.2	0.0	2.3	0.2	2.5	0.3	0.9	0.0	1.9
GhostTownFly	0.4	0.4	0.0	0.0	0.9	0.6	0.0	0.0	0.5	-0.1	0.8	1.1	0.0	0.1
PoznanHall2	0.5	0.7	0.8	0.2	1.0	1.7	0.1	3.2	0.9	1.3	0.4	0.6	0.9	3.7
PoznanStreet	0.9	1.2	0.0	0.0	0.9	1.2	0.0	0.9	0.8	1.7	1.0	0.9	0.1	1.1
UndoDancer	0.2	0.1	0.2	-0.1	0.8	1.0	0.1	0.2	0.8	1.0	1.1	1.0	0.2	0.2
1024×768	0.4	1.0	0.0	0.0	0.4	1.5	0.0	3.0	0.8	2.3	0.3	0.7	0.0	3.0
1920×1088	0.5	0.6	0.3	0.0	0.9	1.1	0.1	1.1	0.8	1.0	0.8	0.9	0.3	1.3
Average	0.5	0.8	0.2	0.0	0.7	1.3	0.0	1.9	0.8	1.6	0.6	0.8	0.2	2.0

**Table 7:** Overall coding time saving compared with the state-of-the-art fast 3D-HEVC algorithms (%).

	CACUP	FMDT	FMD	QTL	ECU	CFM	Fast HTM
Balloons	70	33	68	37	60	40	69
Kendo	64	29	60	35	52	34	64
Newspaper	69	25	71	40	62	42	70
GhostTownFly	73	26	65	42	49	36	68
PoznanHall2	73	35	70	38	51	36	74
PoznanStreet	71	29	64	37	54	28	67
UndoDancer	71	20	64	40	60	41	66
1024×768	68	29	66	37	58	39	68
1920×1088	72	28	66	39	53	35	69
Average	70	28	66	38	55	37	68

### 5.2 Comparison with HTM 16.0 encoders

Table 4 provides coding results of the overall algorithm compared to HTM 16.0 original encoders without any simplifications during mode decision. It can be seen from Table 4 that the overall coding time saving is with the maximum of 73% in "PoznanHall2" and the minimum of 64% in "Kendo". The coding time savings of the proposed method are constant for video sequences with different motion activities: slow object motion sequences like "PoznanHall2" achieve 73% overall coding time saving while high activity sequences like "PoznanStreet" and "Balloons" still can achieve 64–71% coding time saving. It also can be seen from Table 4 that coding time savings are constant for texture video coding and depth map coding, where the texture video coding time and the depth map coding time are respectively reduced by 66% and 74%. As far as coding efficiency is concerned, the proposed algorithm gives a little loss on the central view ("Video 0") but some gains on the dependent view ("Video 2"), which leads to 0.3% BD-rate increase on "Video 0" and achieves 0.1% BD-rate decrease on "Video 2", respectively. The gain on the dependent views is not sufficient to compensate the losses on the central view. Hence, coding losses can be seen in "Video PSNR/Video bitrate", with 0.5% BD-rate increase. The proposed algorithm can reduce bitrate of depth coding and thus achieve a better coding efficiency in "Video PSNR/Total bitrate", with 0.1% BD-rate increase. The proposed algorithm slightly degrades the quality of synthesized views and leads to 0.8% BD-rate increase in "Synth PSNR/Total bitrate", which is caused by the deterioration of the texture video and depth map qualities due to fail to find the optimal CU size or prediction mode for some blocks. Therefore, the proposed algorithm can efficiently reduce complexity of texture video coding and depth coding with a little loss of RD performance compared to the original HTM encoder.

**Fig 9:**
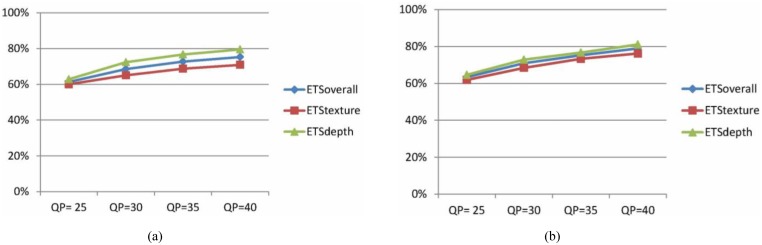
Coding time saving of the proposed method compared to HTM encoders under different QPs. (a) Ballon; (b) GhostTownFly.

Fig 9 shows the overall coding time saving, texture coding time saving and depth coding time saving of the proposed algorithm under different QPs (25, 30, 35, and 40) for two typical sequences "Ballon" and "GhostTownFly". It can be seen from Fig 9 that the proposed algorithm is able to achieve the consistent time saving over a large bitrate range compared to HTM encoders.

**Fig 10:**
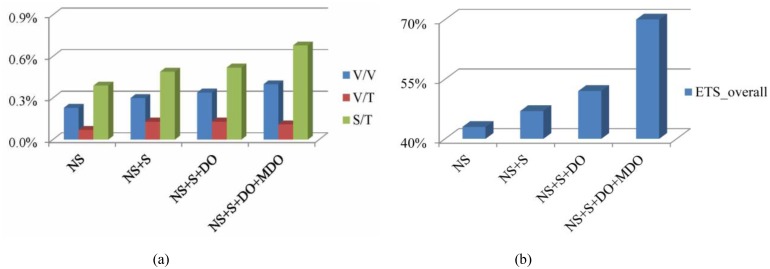
Results of different components of our proposed method in an incremental fashion. (a) BD-rate result; (b) Coding time result.

We also test different components of our algorithms in terms of BD-rate and coding time savings in an incremental fashion in Fig 10, which includes early Non-SPLIT CU determination (NS), early SPLIT CU determination (S), early DE-omitted CU determination (DO) and early ME-DE-omitted CU determination (MDO). This test methodology is chosen intentionally for providing more insight regarding the relative merits of the proposed components and the associated interactions between them. Seven video sequences in Table 3 are used to assess the performance of different components. It can be clearly seen from Fig 10(a) that the performance of the proposed algorithm (either in its entirety or in terms of individual components) has a similar RD performance of the original HTM encoders, leading to 0.4–0.8% BD-rate increase in "Synth PSNR/Total bitrate". As far as the overall coding time saving in Fig 10(b) is concerned, about 43% overall coding time is reduced by "NS" compared to the original HTM encoder. The time savings gradually increase from algorithm "NS" to "NS+S+DO+MDO", which reveals that the addition of the individual component (such as "S", "MDO" and "DO") to "NS" clearly benefits the time savings.

We further shows the experimental results of the proposed overall algorithm in Table 5 as compared to the original HTM encoder with the fast 3D-HEVC tool ("QTL") enabled. As compared to the HTM encoder with "QTL" enabled, the proposed algorithm can further reduce 51% encoder complexity for different sequences with negligible loss of RD performance. The average BD-rate increment is about 0.2% in "Video PSNR/Video bitrate" and 0.1% in "Synth PSNR/Total bitrate".

### 5.3 Comparison with the state-of-the-art fast 3D-HEVC algorithms

In addition to the HTM encoders, the results are compared with the fast 3D-HEVC algorithms, i.e., FMDT [29], FMD [30], QTL [27], ECU [20], CFM [21] and the state-of-the-art fast HTM encoder (Fast HTM), which enable two optional fast tools including QTL and FMDT. All methods are implemented on the same computer for comparison. Tables 6 and 7 respectively show the comparison result in terms of BD-rate and overall coding time saving. FMDT performs good RD performance, but its computation reduction is poor. FMD, QTL, ECU and CFM have good computation reduction at the cost of RD performance. Among these four methods, FMD achieves the largest coding time saving, and QTL yields large coding efficiency degradation in terms of "Synth PSNR/Total bitrate". Our proposed method, CACUP achieves 70% coding time reduction as well as appropriate RD performance with only 0.5%–0.8% BD-rate increase on average. Compared with FMDT, CACUP can achieve better performance on time saving. More than 40% coding time can be further reduced. Meanwhile, the average loss of RD performance is negligible, 0.3% and 0.8% BD-rate increase on "Video PSNR/Video bitrate" and "Synth PSNR/Total bitrate", respectively. Meanwhile, CACUP achieves about 15%–33% overall coding time saving compared to QTL, ECU and CFM with a better RD performance. Compared with our previous work, FMD, CACUP achieves a better RD performance with 0.5% BD-rate decrease on "Synth PSNR/Total bitrate" and 0.2% BD-rate decrease on "Video PSNR/Video bitrate". Furthermore, CACUP achieves more complexity reduction when compared to fast HTM, which is 70% versus 68% in coding time saving. Meanwhile, the RD performance is much better than that of the fast HTM, which is 0.8% compared to 2.0% in BD-rate increase on "Synth PSNR/Total bitrate". The above experimental results indicate that the proposed context-adaptive based CU processing algorithm is efficient for all types of video sequences and outperforms state-of-the-art fast algorithms for 3D-HEVC.

## 6. Conclusion

In this paper, we propose a fast 3D-HEVC encoding algorithm by classifying each CU into five types including DE-omitted CU, ME-DE-omitted CU, SPLIT CU, Non-SPLIT CU and normal CU, and then each type of CU adopts different processing strategies. The proposed algorithm is implemented on the recent 3D-HEVC reference encoder, HTM 16.0. Experimental results show that the proposed algorithm can reduce about 70% computational complexity compared to the original HTM encoder with a negligible loss of RD performance, exhibiting applicability to various types of video sequences. It consistently outperforms 6 state-of-the-art fast 3D-HEVC algorithms with about 4–42% coding time saving or 1.2% BD-rate decrease in "Synth PSNR/Total bitrate".

## Supporting information

S1 File Results of our algorithm compared to HTM. (ZIP)
